# A Review on Colistin Resistance: An Antibiotic of Last Resort

**DOI:** 10.3390/microorganisms12040772

**Published:** 2024-04-11

**Authors:** Aftab Hossain Mondal, Kriti Khare, Prachika Saxena, Parbati Debnath, Kasturi Mukhopadhyay, Dhananjay Yadav

**Affiliations:** 1 Department of Microbiology, Faculty of Allied Health Sciences, Shree Guru Gobind Singh Tricentenary University, Gurugram 122505, Haryana, India; aftab_fahs@sgtuniversity.org (A.H.M.); parbatidn@gmail.com (P.D.); 2 Antimicrobial Research Laboratory, School of Environmental Sciences, Jawaharlal Nehru University, New Delhi 110067, India; kriti.29khare@gmail.com (K.K.); prachika.botany@gmail.com (P.S.); kasturim@mail.jnu.ac.in (K.M.); 3 Department of Life Science, Yeungnam University, Gyeongsan 712-749, Republic of Korea

**Keywords:** antibiotic resistance, colistin resistance, mechanism of resistance, *mcr* genes, prevalence

## Abstract

Antibiotic resistance has emerged as a significant global public health issue, driven by the rapid adaptation of microorganisms to commonly prescribed antibiotics. Colistin, previously regarded as a last-resort antibiotic for treating infections caused by Gram-negative bacteria, is increasingly becoming resistant due to chromosomal mutations and the acquisition of resistance genes carried by plasmids, particularly the *mcr* genes. The mobile colistin resistance gene (*mcr*-1) was first discovered in *E. coli* from China in 2016. Since that time, studies have reported different variants of *mcr* genes ranging from *mcr*-1 to *mcr*-10, mainly in Enterobacteriaceae from various parts of the world, which is a major concern for public health. The co-presence of colistin-resistant genes with other antibiotic resistance determinants further complicates treatment strategies and underscores the urgent need for enhanced surveillance and antimicrobial stewardship efforts. Therefore, understanding the mechanisms driving colistin resistance and monitoring its global prevalence are essential steps in addressing the growing threat of antimicrobial resistance and preserving the efficacy of existing antibiotics. This review underscores the critical role of colistin as a last-choice antibiotic, elucidates the mechanisms of colistin resistance and the dissemination of resistant genes, explores the global prevalence of *mcr* genes, and evaluates the current detection methods for colistin-resistant bacteria. The objective is to shed light on these key aspects with strategies for combating the growing threat of resistance to antibiotics.

## 1. Introduction

Antibiotic resistance poses a significant global risk to public health and its welfare owing to the excessive use of antibiotics together with bacterial evolution. Microorganisms employ various mechanisms to develop resistance to antibiotics as well as other stimulants in the environment. In the presence of antimicrobial agents, bacteria can alter their genetic platform for improved performance as well as to overcome the effects of the antibiotics [1]. The development of antibiotic resistance enables bacteria to outcompete sensitive strains, particularly in selective environments. Moreover, bacteria are increasingly becoming resistant to multiple antimicrobial agents, making it challenging to treat infectious diseases with the limited availability of antibiotics [2]. Colistin, an older antibiotic often referred to as the “last-resort drug,” has been traditionally employed in the management of Gram-negative bacterial infections that are resistant to several antibiotics [3,4]. However, one major problem is that many Gram-negative bacteria now have genes that are resistant to colistin, which reduces the number of effective antibiotics available.

Polymyxin E, another name for colistin, is a polycationic antibiotic renowned for its efficacy against Gram-negative bacteria, particularly *Enterobacteriaceae*. Resistance to colistin primarily arises from chromosomal mutations, which modify the lipid A component of bacterial lipopolysaccharide, reducing its interaction with colistin through cationic substitutions [5]. Thus, the main target of colistin is the lipopolysaccharide found in Gram-negative bacteria’s outer cell membrane. By attaching itself to the lipid A component, the diaminobutyric acid (Dab) residue and the phosphate groups of the lipid A region interact with each other electrostatically, displacing divalent cations (Ca^2+^ and Mg^2+^) and inducing cell death [6,7]. Some bacteria harbor natural resistance mechanisms to prevent the effect of colistin (e.g., *Proteus mirabilis* and *Serratia marcesens*), whereas others acquire resistance (e.g., *Escherichia coli*, *Acinetobacter baumannii*, *Pseudomonas aeruginosa*, *Enterobacter* spp., *Salmonella* spp., and *Klebsiella* spp.) [8]. Several strategies are employed by bacteria to develop resistance to colistin, including the alteration of LPS along with the reduction in its negative charge, the overproduction of efflux pump and capsule formation [9,10,11,12,13]. Colistin resistance in Gram-negative bacteria is associated with the expression of several genes and operons. These include pmrC, pmrE, and the pmrHFIJKLM operon, which directly modify lipopolysaccharide (LPS) [8,14]. Additionally, regulatory systems like PhoP-PhoQ (PhoPQ) and PmrA-PmrB (PmrAB) play crucial roles in governing the PmrAB system [15,16]. Plasmid-mediated *mcr* genes are also implicated, along with acrAB and kpnE, which activate efflux pump systems, and Cpx and Rcs, which up-regulate capsule biosynthesis [17]. Altogether, it helps the bacteria to survive the attack of colistin and become resistant to it for future events.

Concerns have mounted over the misuse and overuse of colistin, leading to increased antimicrobial resistance [18]. As a last-resort antibiotic, the usage of colistin has escalated for the treatment of infections caused by Gram-negative bacteria [19,20,21]. Colistin is commonly used against extensively drug-resistant (XDR), pan-drug-resistant (PDR), and multidrug-resistant (MDR) bacterial infections, which are normally left untreated by other classes of antibiotics [22]. The emergence of colistin resistance complicates treatment options, emphasizing the urgent need for prudent antibiotic use and the development of new antimicrobial agents. Rapidly emerging bacterial resistance to the broadest class of antibiotics has made it hard for us to depend on the development of new antimicrobial agents [1]. Therefore, strategic utilization of older antibiotics is imperative for combating infectious bacterial diseases.

## 2. Importance of Colistin

Colistin belongs to the class of cationic antimicrobial peptides called polymyxins, which comprise non-ribosomal cyclic lipopeptide antibiotics. In clinical medicine, polymyxins are frequently utilized, such as polymyxin B and polymyxin E (colistin). Colistin, initially isolated in 1947 from the Gram-positive *Paenibacillus polymyxa* subsp. *colistinus* bacterium [23], gained widespread use in the 1950s in Japan and Europe in intravenous formulations [24]. Subsequently, in 1959, colistin was authorized by the U.S. FDA to treat MDR Gram-negative bacterial infections including *P. aeruginosa*, *K. pneumoniae*, and *A. baumannii*. For many years, veterinary professionals have widely used colistin to treat and prevent Gram-negative bacterial infections [23]. Its primary application lies in combating Enterobacterial infections, notably those induced by *E. coli* in pig and poultry farming [25,26]. Apart from its common therapeutic and prophylactic purposes in the treatment of infections, colistin is widely employed to promote the growth of pigs [27,28]. In 2018, the Ministry of Agriculture, Forestry and Fisheries categorized colistin as a second-line drug in veterinary medicine due to the emergence of antimicrobial-resistant bacteria from colistin usage in livestock. For the treatment of human Gram-negative bacterial infections, two therapeutically accessible types of colistin are employed: colistin sulfate (CS) and colistimethate sodium (CMS) or colistin methanesulphonate (CMS) [29]. The former, colistin sulfate, an active form, is usually administered orally for digestive tract decontamination and topically for bacterial skin infections. CMS, an inactive prodrug of colistin, is suitable for parenteral, intramuscular, and intraventricular administration due to its lower toxicity compared to colistin sulfate [30]. CMS forms partially sulphomethylated derivatives along with colistin sulfate, the active form of the drug, via hydrolysis. As a dormant prodrug of colistin, CMS by itself shows little or no antimicrobial activity until colistin sulfate is formed [31].

## 3. Mechanism of Action of Colistin against Gram-Negative Bacteria

Colistin primarily targets the outer membrane (OM) of Gram-negative bacteria, specifically the LPS layer. Three domains make up the LPS: the innermost lipid A, the central core oligosaccharide region, and the outermost O-antigen chain [32]. Among these domains, lipid A plays a key role in stabilizing the overall OM structure by rigidly attaching to the fatty acyl chains. Additionally, cations like Ca^2+^ and Mg^2+^ facilitate connections between adjacent LPS molecules, contributing to OM stability [33,34]. Colistin’s antimicrobial activity stems from electrostatic exchanges between the phosphates of lipid A on the bacterial outer membrane and the cationic Dab (diaminobutyric acid) residue on colistin [35]. The Dab residue exhibits three key characteristics: (i) two methylene groups on the Dab side chain; (ii) cationic side-chain groups; and (iii) the particular order of the Dab residues [6]. Furthermore, the incorporation of the lipophilic region of D-Leu6-L-Leu7 (polymyxin E) into the bacterial outer membrane may have an impact on the antibacterial efficacy of colistin [36]. Colistin exhibits antimicrobial efficacy against Gram-negative bacteria through five distinct mechanisms.

### 3.1. The Classical Membrane Lysis Pathway

**Figure 1:**
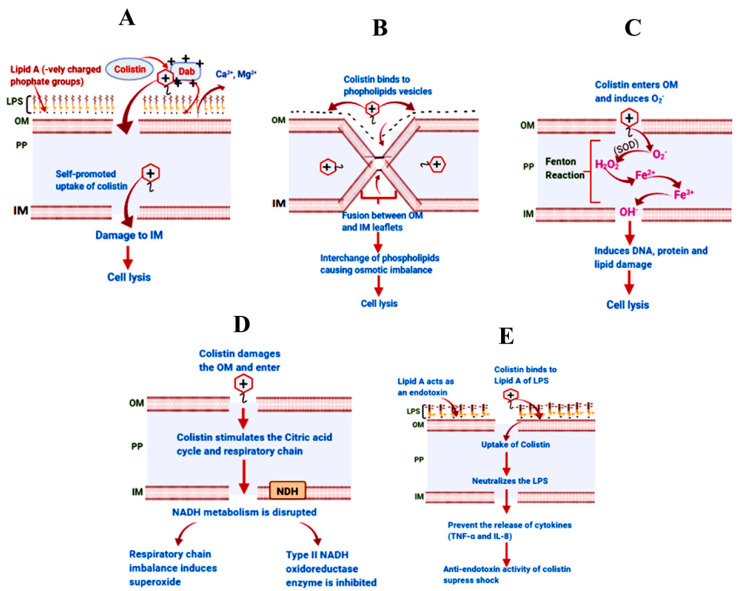
Mechanisms of action of colistin against Gram-negative bacteria. (**A**) Classical membrane lysis pathway; (**B**) vesicle–vesicle contact pathway; (**C**) hydroxyl radical death pathway; (**D**) respiratory enzyme inhibition pathway; (**E**) anti-endotoxin activity of colistin. (Created with BioRender.com).

The most classical mechanism of action is the membrane lysis pathway which is brought about by the electrostatic interaction between negatively charged phosphate head groups on lipid A and positively charged Dab residues on the colistin of the LPS component in Gram-negative bacteria’s outer membrane [6]. Through this interaction, divalent cations, including Ca^2+^ and Mg^2+^, are displaced from the anionic phosphate groups of membrane lipids, destabilizing the LPS [30]. Colistin then enlarges the OM by penetrating the membrane and introducing either the D-Leu6-l-Leu7 segment or its hydrophobic terminal fatty acyl chain, enhancing membrane permeability and facilitating the “self-promoted uptake” of colistin through destabilized areas in the OM formed during its interaction with the LPS [37,38]. Ultimately, the inner membrane (IM) of the phospholipid bilayer is damaged through membrane thinning, which leads to the weakening and loss of the physical integrity of the bilayer causing cell lysis, as shown in Figure 1A [6,11].

### 3.2. Vesicle–Vesicle Contact Pathway

An alternative system contributing to the antibacterial activity of colistin involves vesicle–vesicle contact [39,40]. In this process, colistin facilitates contact between the cytoplasmic membrane’s outer leaflet and the inner leaflet of the outer membrane (OM), through binding to anionic phospholipid vesicles. This interaction promotes the exchange of phospholipids among vesicles, leading to a loss of specificity in phospholipid composition. Ultimately, the disruption of the osmotic balance within the cell occurs, resulting in cell lysis [35], as depicted in Figure 1B.

### 3.3. Hydroxyl Radical Death Pathway

Colistin’s antimicrobial action involves triggering the hydroxyl radical death pathway, as evidenced by previous studies [35,41]. Within Gram-negative bacteria, colistin produces reactive oxygen species (ROS), such as superoxide (O_2_^.−^), peroxide (H_2_O_2_), and hydroxyl radicals (*OH) which cause oxidative stress. Among these, the hydroxyl radical is particularly potent, causing damage to DNA, proteins, and lipids [42]. Upon entering the bacterial cell, colistin prompts the production of O_2_^−^, which is subsequently transformed into H_2_O_2_ by the enzyme superoxide dismutase (SOD). The presence of H_2_O_2_ results in ferric iron (Fe^3+^) being produced by oxidizing ferrous iron (Fe^2+^) and triggers the Fenton reaction, leading to the production of *OH. The oxidative damage caused by these reactive oxygen species to cellular constituents, such as proteins, lipids, and DNA, ultimately culminates in cell death [35]. Importantly, colistin’s efficacy against *E. coli* and *A. baumannii* strains that are not resistant to colistin is attributed to its ability to induce the production of hydroxyl radicals through the Fenton reaction [41].

### 3.4. Respiratory Enzyme Inhibition Pathway

Colistin has recently been discovered to possess a secondary mode of action: the targeting of essential respiratory enzymes within the Gram-negative bacteria’s inner membrane (IM) [43]. Specifically, the Type II NADH oxidoreductase respiratory enzyme is the secondary target of colistin situated in the bacterial electron transport system within the IM [43]. Unlike Type I NADH oxidoreductase, Type II NADH oxidoreductase, also known as “alternate NADH oxidoreductase,” does not actively pump protons across the IM [44]. Colistin inhibits the Type II NADH oxidoreductase by stimulating the respiratory chain and thus accelerating its consumption [45]. This inhibition disrupts the bacterial electron transport system, impairing respiratory function and compromising bacterial viability.

### 3.5. Anti-Endotoxin Activity of Colistin

Colistin’s interaction with the lipid A component of LPS in bacteria that are Gram-negative extends to its anti-endotoxin activity [30,46]. By binding to the lipid A, colistin effectively targets the endotoxin, neutralizing LPS molecules and indirectly inhibiting endotoxin activity. Endotoxins can trigger inflammatory responses upon infection, releasing cytokines like interleukin-8 (IL-8) and tumor necrosis factor-alpha (TNF-α), which may induce shock [46,47].

## 4. Molecular Mechanisms of Colistin Resistance

**Figure 2:**
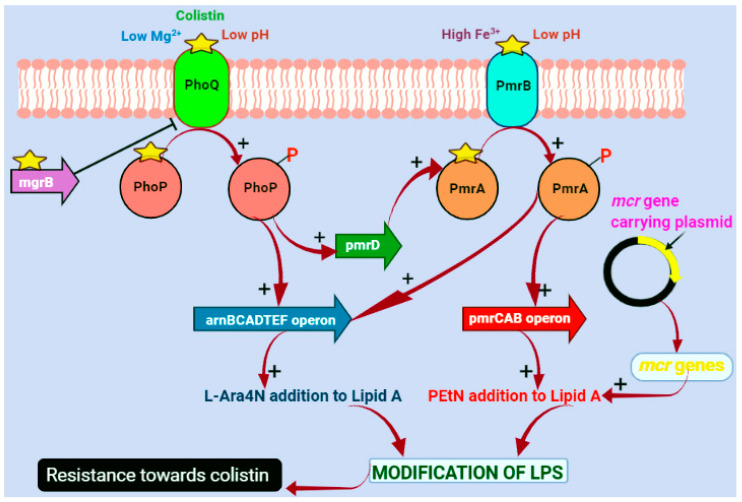
Mechanisms of colistin resistance in Gram-negative bacteria. (created with BioRender.com).

Colistin resistance poses a significant challenge due to the limited availability of effective antibiotics to combat resistant pathogens [48]. The rise of antibiotic-resistant bacterial strains is largely attributed to the increased and inappropriate use of antibiotics. Some Gram-negative bacteria such as *P. aeruginosa*, *A. baumannii*, and *K. pneumoniae* have acquired resistance to colistin, while others, like *Serratia* spp., *Burkholderia* spp., and *Proteus* spp., exhibit natural resistance [8]. These bacteria employ various strategies, such as LPS alterations, the utilization of efflux pumps, and capsule formation, to develop resistance to colistin (Figure 2).

### 4.1. Chromosomal-Mediated Colistin Resistance

One of the primary mechanisms of chromosomal-mediated colistin resistance involves the modification of the lipopolysaccharide (LPS) layer through cationic substitution, as elucidated by Poirel L. et al. (2017) [23]. Colistin’s antibacterial activity hinges on its electrostatic interaction with the negatively charged LPS layer. Bacterial cells can mitigate this interaction by altering the lipid A to lower the net negative charge, thereby enhancing colistin resistance. This modification entails the inclusion of phosphoethanolamine (PEtN) and 4-amino-4-deoxy-L-arabinose (L-Ara4N) in the lipid A LPS moiety [28,49]. This addition of cation moieties on LPS is accomplished using two complementary regulatory systems (TCSs). Alterations within these regulatory systems and perhaps their associated genes result in constitutive activation and upregulation, thereby producing an increased number of cationic moieties in LPS. This process inhibits the activity of colistin by lowering the outer membrane’s (OM) net negative charge [29].

The two-component systems (TCSs) PmrA-PmrB (PmrAB) and PhoP-PhoQ (PhoPQ) stand out as extensively studied regulators that contribute significantly to colistin resistance [50,51]. Among these, the PmrA-PmrB system, encoded by the pmrCAB operon and activated by PhoP-PhoQ, serves as a key regulator supporting the lipid A modification in Gram-negative bacteria [52]. Under low-Mg^2+^-growth conditions or exposure to sub-lethal concentrations of cationic antimicrobial peptides like polymyxin, the system of PhoP-PhoQ is activated. The sensor kinase PhoQ phosphorylates the response PhoP regulator, which subsequently uses the PhoP-activated PmrD protein to activate PmrA-PmrB [52,53,54,55,56,57]. In response to extra-cytoplasmic Fe^2+^ and acidic pH, the enzyme sensor kinase PmrB facilitates the phosphorylation of the response regulator PmrA. This results in PmrA-activated genes’ transcription, such as arnBCADTEF and pmrE genes, which facilitate LPS modification by adding L-Ara4N and repressing PmrA-repressed genes [58,59]. These modifications orchestrated by the PmrA-PmrB and PhoP-PhoQ systems cause the negative charge of LPS to decrease, thereby preventing electrostatic interaction with polymyxin. Chromosomal mutations regulating TCSs have been connected to resistance to colistin among various Gram-negative bacteria, including *K. pneumoniaea*, *P. aeruginosa*, *Salmonella* spp., *A. baumannii*, *E. coli*, and *Enterobacter* spp., have shown to acquire resistance towards colistin via chromosomal mutations through the regulation of TCSs [1]. Gram-negative bacteria that are resistant to colistin can also arise through the action of multidrug efflux pumps. For instance, the MexAB-OprM efflux pump in *P. aeruginosa* confers resistance to colistin by enhancing mexAB-oprM expression upon exposure to the antibiotic [60,61]. Since the association between polymyxin and LPS is essential for the bactericidal action of polymyxin, the entire loss of LPS can also lead to the elevation of colistin resistance as reported against colistin in *A. baumannii* [62,63].

### 4.2. Plasmid-Mediated Colistin Resistance

Prior to 2015, chromosomal alterations were thought to be the main cause of colistin resistance in Gram-negative bacteria, particularly affecting genes and operons involved in lipid A biosynthesis. These mutations, leading to cationic substitutions in the lipid A lipopolysaccharide (LPS) component, led to reduced affinity for colistin, thus decreasing the antibiotic’s efficacy. However, in 2016, a pivotal discovery emerged with the identification of *E. coli* strains from pigs found to harbor the first plasmid-mediated colistin resistance gene, *mcr*-1, isolated from China [15]. The mobile colistin-resistant genes or *mcr* are the plasmid-borne genes that account for colistin resistance via horizontal gene transfer (HGT). This MCR, coded by the *mcr* gene that is a member of the phosphoethanolamine transferase family, catalyzes the addition of PETN (phosphoethanolamine) towards the lipid A LPS moiety. This modification decreases the binding affinity of colistin to the bacterial membrane, thus reducing its antimicrobial activity [64]. The HGT of this plasmid-borne gene *mcr*-1 can be promptly disseminated among various Gram-negative bacteria, conferring colistin resistance at a broader scale and reaching up to the human food chain, thereby becoming a greater concern for public health. As per previous research conducted by Poirel et al. (2017), it was found that the presence of the *mcr-1* gene results in a 4- to 8-fold increase in the minimum inhibitory concentrations (MICs) of colistin in *E. coli* [23]. This underscores the *mcr*-1 gene’s ability to confer colistin resistance autonomously in *Enterobacteriaceae* members and *E. coli*, without requiring further resistance mechanisms. Following its initial discovery in China, the *mcr*-1 gene has been identified worldwide in various Gram-negative species of bacteria. Notably, there has been a notable frequency of the *mcr*-1 gene in isolates of *E. coli* from domestic animals, particularly pigs and chickens, which are recognized reservoirs for *E. coli* with the *mcr*-1 gene in them [65]. The ubiquity of resistant isolates in livestock highlights the widespread application of colistin to treat illnesses brought on by Gram-negative bacteria in animal care. Additionally, MCR-1 has been observed to provide resistance to lysozyme, further enhancing bacterial survival [66]. Despite the elucidation of the MCR-1 catalytic domain’s crystal structure [67], the substrate-binding sites of MCR-1 remain undetermined. Understanding these sites is crucial for comprehending the overall reaction catalyzed by this protein. Consequently, the lack of a well-defined mechanism for the plasmid-mediated resistance of colistin impedes our understanding of the *mcr*-1 gene’s transmission dynamics through horizontal gene transfer (HGT). 

## 5. Dissemination of Colistin Resistance (HGT: Transformation, Transduction, Conjugation)

The plasmid-mediated *mcr*-1 gene was first reported in *E. coli* from China in 2016. Then, a large number of studies reported various variants of *mcr* genes in Gram-negative bacteria from all across the world [68,69]. The increasing prevalence of plasmid-mediated *mcr* genes within Gram-negative bacteria may be due to selective environmental pressure [70]. Hence, there are more than 25 variants of the *mcr*-1 gene, all of which differ from one or two of the amino acids from *mcr*-1 [71,72,73,74]. Surprisingly, there are also other members of the *mcr* gene family besides *mcr*-1, and to date, ten different *mcr* genes have been reported so far (*mcr*-1 to *mcr*-10) [15,16,75,76,77,78,79,80,81].

This global prevalence and dissemination of colistin-resistant determinants have threatened the therapeutic effectiveness of colistin against multidrug-resistant Gram-negative bacteria. The greater risk because of *mcr* gene dissemination arises from horizontal transmission. The spread of plasmid-mediated colistin-resistant determinants is mostly through animals and food markets because of the broad usage of colistin in veterinary medications [82]. Multiple plasmid types are associated with the spread of *mcr*-1, including IncX4, IncHI1, IncHI2, IncP, IncI2, and IncY, among which IncI2 and IncX4 are the most prevalent [81]. IncI2 and IncX4 are together considered “epidemic” plasmids due to their carrying capability and global spread of the *mcr*-1 gene among *Enterobacteriaceae* from animal as well as human sources [83,84]. This molecular flexibility of the *mcr*-1 gene not being associated with a specific incompatibility group of plasmids has been considered a cause of the global dissemination of the gene [85]. The *mcr*-1 gene was originally discovered in the IncI2 plasmid named pHNSHP45 (64105 bp) [86,87]. Subsequently, numerous plasmids, including IncI2, IncFI, IncHI2, IncX4, IncFII, IncFIB, IncK2, IncP, IncF, IncY, IncN, and IncQ, have been found to contain the *mcr* gene [15,77,86,88]. The conjugative property of the plasmid backbone primarily facilitates the widespread distribution of the *mcr* gene in Gram-negative bacteria [81]. The mobility of the *mcr* gene is accompanied by a specific insertion sequence (IS) element within the mobile genetic elements, i.e., transposons. It has been reported that the *mcr*-1 gene in IncHI2 and IncI2 plasmids is flanked by ISApl1 insertion sequences in one or two copies, while the IncX4 plasmid is flanked by two copies of *ISApl1*, located upstream to the gene [15,89]. This flanking by insertion sequences in the *mcr*-1 gene contributes to its transposition activity. The presence or absence of this IS element interrelates when the *mcr*-1 gene is modified for a different host. This means that the presence of two copies of *ISApl1* shows a current acquisition of this IS element, whereas the absence of *ISApl1* or the presence of only one copy reveals its earlier adaptation in the host [90]. Therefore, for the mobility and stabilization of *mcr*-1-bearing plasmid, the loss of a copy of the IS element from the transposon occurs via transposition or non-homologous recombination events, which are ultimately responsible for colistin resistance [23,89].

Apart from *mcr*-1, the distribution of the other *mcr* gene family members has also been associated with IS elements. For instance, the *mcr*-2 gene, which bears resemblance to *mcr*-1, is flanked by an IS element from the superfamily IS1595, which is situated upstream of *mcr*-2 [88]. Upstream of the nimC/nimA-*mcr*-3 gene, the TnAs2 transposon is present, while the IS element ISKpn6 is situated upstream of the ColE10-type *mcr*-4 plasmid. A Tn3-family transposon on a ColE-type plasmid contains the *mcr*-5 gene [80]. Altogether, these plasmid-harboring *mcr* genes have revealed an alternative scenario of colistin resistance via the dissemination of resistant determinants, thus creating a problematic situation in relation to public health. Recent studies have revealed that some of the plasmid-harboring *mcr* genes encode antibiotic-resistant genes (ARGs) like NDM-1, NDM-5, KPC-2, and KPC-3 along with colistin-resistant genes [81,91,92,93]. The coexistence of *mcr* genes with ARGs, particularly carbapenemases, within the same conjugative plasmid, leads to the co-selection of isolates harboring the *mcr* plasmid, facilitating its widespread dissemination [23]. This co-occurrence poses a significant challenge to therapeutic options available to cure bacterial infections.

## 6. Global Prevalence of *mcr*-Mediated Colistin Resistance (Environmental and Clinical Isolates)

The increasing prevalence and dissemination of Gram-negative bacteria (GNB) resistant to colistin is leading to an eventual massive global crisis. GNB utilize a plethora of mechanisms to develop resistance against the drugs of last resort as discussed previously in this article with plasmid-located *mcr* gene-mediated resistance being one of the recently studied and a major cause of concern because of its horizontal transferability. To date, as many as 10 different *mcr* genes (*mcr*-1 to *mcr*-10) have been determined and reported [29,94,95]. The mechanism by which resistance is conferred against the colistin drug, i.e., PEtN transfer, is conserved across all these different allelic variants of the *mcr* gene and they share some conserved amino acids with each other. They are supposed to have a different genetic origin because of the variability in the sequence similarity with one another. Comparisons of their sequences reveal variations in sequence similarity among them. Specifically, *mcr*-1 shares varying degrees of amino acid sequence identity with other alleles: *mcr*-2 (81%), *mcr*-3 (34%), *mcr*-4 (33%), *mcr*-5 (31%), *mcr*-6 (82%), *mcr*-7 (29%), and *mcr*-8 (31%) [96]. These variants of *mcr* genes are found in many different species of GNB. About 91% of all *mcr*-positive isolates are *E. coli*, with *S. enterica* coming in second with 7% and *K. pneumoniae* with 2%; the frequency of the *mcr* gene in different species of GNB like *Cronobacter sakazakii*, *Shigella sonnei*, *Moraxella*, *P. mirabilis*, and other *Enterobacter* species is approximately just 0.2% [95].

The overall average prevalence of *mcr* genes is 4.7% worldwide [97]. The prevalence of *mcr*-positive colistin-resistant *E. coli* is 6.51% globally [68]. As per the 2023 data from the National Database of Antibiotic Resistant Organisms, 3.6% of *mcr*-1 and 1.1% of *mcr*-9 reported in Enterobacterales, 4.5% of *mcr*-3 in *Aeromonas* spp., and 0.2% of *mcr*-10 in *Enterobacter kobei* from various country throughout the world [98].

### 6.1. The mcr-1 Gene

*mcr*-1 gene-mediated colistin resistance has been reported to be present in clinical and environmental isolates for a long time, dating back as early as in pathogens isolated from poultry in the 1980s in China, from veal calves in 2005 in France, and from the clinical blood sample from a patient with hematologic oncology in 2011 in Hungary, but it was first characterized only recently by Yi Yun Liu et al. from China [15,94,99,100]. This first study published by Liu et al. highlighted the growing colistin resistance caused by the *mcr*-1 gene in the members belonging to the *Enterobacteriaceae* family isolated from the samples of human clinical isolates, food animals, and retail meat collected between 2011 and 2014. This gene contains 1626 bp and they observed that colistin-resistant *E. coli* was isolated from pigs and resistance was spreading at a fast rate because the bacteria utilized the horizontal gene transfer mechanism to spread the resistance amongst other members/species rapidly. The frequency at which the *mcr*-1 gene-containing plasmid was mobilized to a recipient cell was 10^−1^ to 10^−3^ cells through the process of conjugation. This plasmid was also seen to be maintained in *K. pneumoniaea* and P. aeruginosa, showing the possible inter-specific transmission of the plasmid [15]. After this first characterization study, several studies were published from 61 countries across 6 continents showing the pandemic spread of *mcr*-1 gene-mediated colistin resistance and its similar genetic variants in members belonging to the *Enterobacteriaceae* family, having a diverse origin [70,81,88,101,102,103,104]. One study showed the *mcr*-1 gene as being present in a virulent pathogen, namely *Vibrio parahaemolyticus*, belonging to a non-*Enterobacteriaceae* family isolated from the shrimp sample that was collected from Hong Kong [105].

Now, more than 25 different variants of the mcr-1 gene have been reported, differing from mcr-1 by one or two amino acid changes [73,74].

The *mcr*-1 gene exhibits a notable prevalence among *Enterobacteriaceae* strains isolated from human specimens [96]. A comprehensive global surveillance initiative, INFORM (International Network for Optimal Resistance Monitoring), was conducted between 2014 and 2016, and systematically collected colistin-resistant *Enterobacteriaceae* samples from various regions worldwide. Screening these samples revealed the existence of plasmid-borne *mcr* genes in 29 isolates (3.2%), originating from 15 different countries. Of these, the *mcr*-1 gene or its variants was present in 24 isolates [106]. This report underscores the widespread prevalence of *mcr* gene-mediated colistin resistance in bacteria that are Gram-negative, emphasizing the significance of vigilant monitoring to combat the emergence of novel resistance mechanisms against commonly used antibiotics.

### 6.2. The mcr-2 Gene

The *mcr*-2 gene, containing 1617 bp and a phosphoethanolamine transferase activity, was present in IncX4 plasmid and was, for the first time, reported to be highly prevalent in bovine and porcine colistin-resistant *E. coli*, collected from Belgium. Evidence from phylogenetic analysis/studies revealed that this gene was distinct from *mcr*-1 and shared 76.74% similarity with it. It was co-harbored with a lipid phosphatase gene, which shared a strong homology with the phosphatase gene present in *Moraxella* spp., thereby suggesting its possible origination from *Moraxella catarrhalis* [16]. In another study published in Belgium, the *mcr*-2 gene was seen to be present for the first time in 0.95% of all the *Salmonella* strains isolated from retail meat samples [107]. In a recent study published in Bangladesh, three *E. coli* strains isolated from poultry and two from street foods have been shown to be harboring the *mcr*-1 and *mcr*-2 genes, respectively. This study underscores the significant prevalence of *E. coli* strains resistant to colistin in food samples, raising concerns about the potential transfer of resistance to humans [108]. A recent study conducted in India, focusing on Gram-negative bacteria isolated from ocular infections, revealed alarming findings. Approximately 40% of the total isolates exhibited colistin resistance with 6.25% and 25% of the isolates harboring plasmids containing the *mcr*-2 and *mcr*-1 genes, respectively [109]. These results highlight the critical requirement for enhanced surveillance and control measures to address the escalating threat of colistin resistance in environmental and clinical settings. A study published in China has identified 33 novel *mcr*-2 gene variations (*mcr*-2.3 to *mcr*-2.35) isolated from pigs and poultry having 95.9–99.9% nucleotide sequence similarity among themselves and 95.8–98% sequence similarity with the first *mcr*-2 gene identified in the year 2016 in Belgium [110]. 

### 6.3. The mcr-3 Gene

The *mcr*-3 gene, spanning 1626 bp, was initially identified in *E. coli* isolated from pigs, exhibiting 47% and 45% nucleotide similarities with the *mcr*-2 and *mcr*-1 genes, respectively [76]. This was found on a conjugative plasmid alongside 18 other resistance genes. In a recent study from Baghdad, environmental and clinical samples collected from 2016 to 2018 were screened for colistin-resistant *A. baumannii* isolates. Remarkably, 67.8% of all the isolates resistant to colistin harbored the *mcr*-3 gene [111]. In another study published in Vietnam, the *mcr-3* gene was reported to be present in 3% of the meat-derived colistin-resistant ESBL- or AmpC-producing *E. coli* [112]. Intriguingly, as many as 30 different genetic variants of the *mcr-3* gene have been reported to date. Subsequently, many reports have been published from several global locations indicating the presence of different variants of the *mcr*-3 gene along with *mcr*-1 on the same hybrid plasmid, which might raise serious public health concerns [113,114]. Therefore, surveillance is needed to conduct risk assessments and investigate the effects of the co-existence of different variants of *mcr* together. The *mcr*-3 gene ranks as the second most prevalent gene granting Gram-negative bacteria tolerance to colistin after *mcr*-1. Wang et al. revealed the existence of a *mcr*-3 gene variation (*mcr*-3.6) in colistin-resistant ESBL-producing *Aeromonas veronii* isolated from chicken feces. The transfer of this variant was mediated through a novel transposon, Tn6518, rather than a plasmid, raising additional concerns [115]. In a different current study, Sun et al. documented the global clonal dissemination of multidrug-resistant ST34 *Salmonella enterica serotype* Typhimurium and monophasic variants harboring the *mcr*-3 gene alongside blaCTX-M-55 and qnrS1. This underscores the potential transmission of *mcr*-3 along with other antibiotic resistance genes via mobile genetic elements [116]. Vigilant monitoring and investigation are essential to address the evolving landscape of colistin resistance and its associated genetic determinants.

### 6.4. The mcr-4 Gene

The *mcr*-4 gene, spanning 1626 bp, was initially identified in *S. enterica*, particularly in a monophasic *serovar* Typhimurium isolate from a pig at slaughter in Italy, and in *E. coli* strains from Spanish and Belgian piglets, indicating its significant dissemination across Europe [78]. A ColE10 plasmid of 8749 bp carried this gene, and its protein product shared 34%, 35%, and 49% amino acid sequence similarity with MCR-1, MCR-2, and MCR-3, respectively. A bacterial species is thought to be the source of this gene primarily inhabiting aquatic environments, such as *Shewanella* sp. A retrospective study characterizing antibiotic resistance patterns and genes in pigs raised on Spanish farms from 1998 to 2018 revealed a notable rise in colistin-resistant *E. coli* isolates during the 2011–2014 period. Among these isolates, 13% harbored the *mcr*-4 gene, while 7% and 3% contained *mcr*-1 and *mcr*-5, respectively [117]. In another study, the *mcr*-4 gene was most prevalent among Gram-negative bacteria resistant to colistin isolated from vaginal swabs of women, with 12.7% of isolates testing positive for *mcr*-4, followed by 1.5% positive for *mcr*-2 and *mcr*-3, and only 0.7% positive for *mcr*-1 and *mcr*-5 [118]. To date, six different genetic variants of the *mcr*-4 gene have been reported (*mcr*-4.1 to *mcr*-4.6). In a research project carried out in Spain, strains of *E. coli* causing post-weaning diarrhea on extensive pig farms were isolated, with a surprising 76.9% showing colistin resistance. Among these resistant isolates, the majority (72.8%) harbored the *mcr*-4 gene, which was also found to coexist with *mcr*-1 in a few isolates. This report underscores that the *mcr*-4 gene is highly prevalent in Spain [119]. These findings highlight the urgent need for surveillance and control measures to address the uprising prevalence of colistin resistance as well as its associated genetic determinants.

### 6.5. The mcr-5 Gene

The *mcr*-5 gene consists of 1644 base pairs and is included in a 7337-base-pair transposon residing on a plasmid of the ColE type and belongs to the Tn3 family. It was initially characterized in *Salmonella* Paratyphi BdTa+ isolated in Germany from poultry [80,110]. The protein product of this gene, comprising 547 amino acids, shares sequence similarities of 36.11%, 35.29%, 34.72%, and 33.71% with MCR-1, MCR-2, MCR-3, and MCR-4 proteins, respectively. Numerous reports from various parts of the globe suggest the global dissemination of this gene and its variants [120]. The *mcr*-5 gene was first characterized in a colistin-resistant *A. hydrophila* isolate, with a colistin MIC value of 4mg/mL, isolated from pig fecal samples collected in rural areas of China [121]. In another study from China, the frequency of the genes *mcr*-4 and *mcr*-5 in nasal and anal swabs collected from pigs and poultry was investigated. The frequency of the *mcr*-5 gene was higher in swine swabs (33.1% of all isolates) compared to poultry swabs (5.6%) [122]. Additionally, the *mcr*-5 gene was found in 24% of samples collected from poultry farms in Paraguay. The plasmids carrying the *mcr*-5 gene varied in size from ~80 to 200 kbps and exhibited phenotypic resistance to multiple antibiotics besides colistin, and 82% of the plasmids were capable of utilizing the conjugation mechanism for horizontal transferability [123]. To date, four different genetic variants of the *mcr*-5 gene have been reported (*mcr*-5.1 to *mcr*-5.4).

### 6.6. The mcr-6 Gene

AbuOun et al. identified a genetic variant of the *mcr*-2 gene present in *M. pluranimalium*, pigs’ isolate, which they later named *mcr*-6.1 in Great Britain [75]. This *mcr*-6 gene contains 1617 base pairs and exhibits phosphoethanolamine-lipid A transferase activity. To date, only one variant of this gene, *mcr*-6.1, has been reported. Remarkably, *mcr*-6 is the only variant of the mobile colistin-resistance gene that has not been reported from China yet [97].

### 6.7. The mcr-7 Gene

This gene, containing 1620 base pairs, was initially detected in poultry strains of *K. pneumoniae* that were isolated in China [77]. The protein product of this gene shares the highest amino acid sequence similarity with MCR-3, reaching 70% among all other *mcr* variants. It was found to be located on a conjugative plasmid capable of transferring from *K. pneumoniae* to *E. coli*, resulting in an eightfold increase in the minimum inhibitory concentration (MIC) of the drug colistin. In a study investigating the presence of antibiotic resistance genes (ARGs) in environmental and fecal samples in 2019, which were collected from a zoo in Brazil, the detection of the *mcr*-7.1 gene was conducted in water samples collected from the surroundings of the alligator. This finding underscores the potential role of zoos as reservoirs of ARGs, highlighting the need for vigilant monitoring to prevent further spread [124]. Another study from Germany discovered the identification of *mcr*-7, along with *mcr*-3, in high prevalence in samples collected from municipal wastewater [125]. To date, only one genetic variant of *mcr*-7 has been described, namely *mcr*-7.1.

### 6.8. The mcr-8 Gene

This gene, comprising 1698 base pairs and located on a conjugative plasmid, was first identified in a *K. pneumoniae* strain resistant to both colistin and carbapenem that was obtained from samples of human sputum and pig feces in China [81,126]. The protein encoded by the *mcr*-8 gene exhibited amino acid sequence similarities of 31.08%, 30.26%, 39.96%, 37.85%, 33.51%, 30.43%, and 37.46% with MCR-1, MCR-2, MCR-3, MCR-4, MCR-5, MCR-6, and MCR-7, respectively. To date, five different genetic variants of *mcr*-8 have been reported, including the recently characterized variant, *mcr*-8.5, in 2020. In a recent study from Bangladesh, *mcr*-8.1 was found to be present on a stable plasmid with a 0.3% frequency rate in multidrug-resistant *K. pneumoniae* strains, which were recovered from sick patients in the biggest public hospital. This finding raises concerns regarding the potential dissemination of this gene across South Asia [127]. In another study, the *mcr*-8 gene variant (*mcr*-8.3) was found to co-occur with *mcr*-3 gene variants (*mcr*-3.21, *mcr*-3.26, and *mcr*-3.28) in strains of *K. pneumoniae* identified from healthy human stool samples collected in Thailand and Laos [128]. Furthermore, cloaca samples collected from commercial poultry in China revealed that various *Raoultella* species harbored the *mcr*-8 gene, with the *mcr*-8.4 variant specifically present in *R. ornithinolytica*. The increased transferability of the plasmid containing this gene, or its variants, upon entering *E. coli*, along with its co-transfer with other antibiotic resistance genes, raises concerns about the rapid dissemination of this gene among *Enterobacteriaceae*.

### 6.9. The mcr-9 Gene

This gene, characterized only recently in 2019, was found in an *S. enterica* strain of serotype Typhimurium that was first isolated from a patient in Washington State in 2010. When the predicted protein structures of MCR-9 and other MCR homologues were compared, it was found to share a high degree of structural similarity with MCR-3, MCR-7, and MCR-4. The *mcr*-3.17 allele exhibited the highest MCR-9 identification of amino acids at 64.5% [79]. In just a year, numerous reports from various regions of the globe have provided evidence of the global spread of this gene. MCR-9 is the second most widely distributed gene globally after MCR-1 and has been reported in 40 countries across 6 continents [101]. It has been found in various *Enterobacteriaceae* species collected from diverse origins, including environments, animals, food, and humans [129]. In a study from Egypt, the *mcr*-9 gene was found to co-harbor with blaVIM-4 in multidrug-resistant *Enterobacter hormaechei* isolated from a patient’s sputum. Another study revealed that the *mcr*-9 gene observed to coexist with blaNDM-1 in multidrug-resistant *Enterobacter cloacae* was identified from a patient with a blood infection [130]. To date, three different genetic variants of this gene have been reported, namely *mcr*-9.1 to *mcr*-9.3.

### 6.10. The mcr-10 Gene

Recently, significant discoveries have been made regarding the appearance and spread of new genes resistant to colistin, particularly *mcr*-10, signaling potential challenges in clinical settings. In a comprehensive screening of 941,449 genomes of bacteria from the GenBank database, *mcr*-10-harboring isolates were identified, primarily among clinically threatening *K. pneumoniae* ST11 strains. Notably, these isolates co-harbored *mcr*-10 and *mcr*-8 genes, displaying reduced susceptibility not only to polymyxins but also to tigecycline. This discovery underscores the sporadic distribution of *mcr*-10 across various sources worldwide, with a notable prevalence in human-related contexts, raising serious clinical concerns [131]. In another study, researchers discovered that the unique IncFIB-type plasmid carried by the *E. coli* strain EC2641 carried the mobile colistin resistance gene, or *mcr*-10. This finding is particularly alarming as it demonstrates the capacity of *mcr*-10 to confer resistance upon transformation. Using phylogenetic analysis, it was found that *E. coli* EC2641 was grouped with clinically significant, multidrug-resistant disinfectants and colistin from the United States, suggesting the potential global dissemination of *mcr*-10 [132]. Furthermore, investigations into colistin-resistant *Enterobacter* spp. isolates revealed the absence of *mcr*-1 to *mcr*-8 genes but identified *mcr*-9 and *mcr*-10 at rates of 8.4% and 12.6%, respectively. Remarkably, all *mcr*-9- and *mcr*-10-positive *Enterobacter* isolates belonged to the *Enterobacter cloacae* complex (ECC), consisting primarily of plasmid-based genes. Notably, the plasmids harboring *mcr*-9 seemed to have a conserved backbone, whereas the plasmids harboring *mcr*-10 showed variability in their backbone, indicating potential differences in transmission dynamics [133]. Additionally, with the highest nucleotide identity (79.69%) with *mcr*-9, a new *mcr*-10 variant was reported on an IncFIA plasmid of a clinical strain of *E. roggenkampii*. Intriguingly, *mcr*-10, along with *mcr*-3 and *mcr*-1, has been incorporated into the chromosomes of certain bacteria, facilitated by ISs and integrative conjugative elements, further complicating the landscape of colistin resistance [134].

## 7. Phenotypic and Molecular Detection Methods of Colistin-Resistant Gram-Negative Bacteria

The emerging resistance of GNB to colistin, the drug of last resort, is very alarming. There need to be proper detection methods that can help in the rapid and reliable identification and characterization of resistant bacteria isolated from clinical or environmental settings [135,136]. Here, we have tried to summarize the different phenotypic as well as molecular methods that are most commonly used to detect colistin-resistant GNB.

### 7.1. Screening Resistant Isolates Using Selective Media

Microbiologists frequently employ selective agar-based media to isolate specific bacteria from complex samples obtained from environmental or clinical sources. This method aids in the initial screening of resistant bacterial isolates and makes it easier to identify all resistant strains, including those that have resistance mechanisms that are novel or unknown. However, this detection method has a significant drawback due to its lengthy turnaround time, typically ranging from 18 to 48 h, and the limited insight it provides into the mechanism of resistance. Among the most commonly used media for detecting polymyxin (colistin) resistance are LBJMR medium, SuperPolymyxinTM, and CHROMagar COL-APSE. The composition of these media is such that they include amphotericin B, which inhibits the growth of fungi, and vancomycin/daptomycin, which inhibits the growth of bacteria that are Gram-positive and low concentrations of colistin to detect only colistin-resistant Gram-negative bacterial isolates, even the strains that have acquired resistance as well as inhibit the susceptible strains.

➢ Super Polymyxin: It is a widely utilized screening medium, developed by ElitechMicrobio in Signes, France. It contains a lower concentration of colistin, specifically 3.5 μg/mL, which enables the straightforward identification of isolates resistant to colistin that have low minimum inhibitory concentration (MIC) values, whether the resistance is intrinsic or acquired [137,138]. In addition to colistin, SuperPolymyxin contains 5 μg/mL of amphotericin B, 10 μg/mL of daptomycin, and EMB powder. The EMB powder produces a distinct metallic green reflection and suppresses the growth of Gram-positive bacteria with selectivity.
➢ CHROMagar COL-APSE: It is an agar-based selective medium developed subsequent to SuperPolymyxin, offering a broader target spectrum [139]. This medium incorporates colistin sulfate and oxazolidinone antibiotics. Its chromogenic properties enable the efficient differentiation of *Enterobacteriaceae* as well as colistin-resistant Gram-negative non-fermenters.
➢ LBJMR (Lucie-Bardet-Jean-Marc-Rolain) Medium: This recently developed medium offers versatility by detecting colistin-resistant *Enterobacteriaceae*, Gram-negative non-fermenters, and vancomycin-resistant enterococci (VRE) [140]. Enhanced sensitivity and specificity were observed with a purple agar base supplemented with bromocresol purple and glucose compared to other combinations. Yellow colonies of *Enterococci* and *Enterobacteriaceae*, varying in size, are observable against the purple background. Additionally, this medium can facilitate the detection of pathogens in patients diagnosed with cystic fibrosis.

### 7.2. Determination of MIC

The lowest dose at which a medication, like colistin, can be used to prevent observable bacterial growth is known as the minimum inhibitory concentration or MIC. It is a widely used test in microbiological laboratories to assess bacterial susceptibility to various antibiotics. To determine MIC, a pure culture is required, and samples directly collected from the environment or clinical settings cannot be used. BMD (Broth Microdilution assay) is the most commonly employed method for determining bacterial susceptibility to polymyxin, suggested by both the European Committee on Antimicrobial Susceptibility Testing (EUCAST) and the Clinical and Laboratory Standards Institute (CLSI). It is advised to perform this test using non-polystyrene plates and replace the often-used colistimethate version of colistin in human medications with sulfate salts of the drug. Additionally, polysorbate 80 should be avoided [141,142]. Both CLSI and EUCAST have established breakpoints for colistin for different organisms. However, the BMD process is laborious, time-consuming, and may yield varying results upon repetition. Based on BMD, several commercial systems have been developed to determine the MIC easily in less time like the Sensititre system, Vitek2, BD Phoenix, etc.

- The Sensititre system developed by Thermo Fisher Scientific (Waltham, MA, USA) has a customizable plate layout with 96 wells containing antibiotics. All the steps including inoculation, incubation, and reading can be automated [143].
- The Vitek 2 system developed by BioM’erieux, Marcy l’Etoile, France uses 64 wells containing dehydrated antibiotics and other reagents and is semi-automated [143].
- BD Phoenix developed by Becton Dickinson, Le Pont de Claix, France uses an 84-well plate and an oxidation-reduction indicator to determine the antimicrobial susceptibility in 6–16 h.

### 7.3. Matrix-Assisted Laser Desorption-Ionization Time-of-Flight Mass Spectrometry (MALDI-TOF MS)

MALDI-TOF, a long-standing tool in proteomics, has recently garnered significant attention from microbiologists due to its promise in identifying bacterial phylum, species, sub-species, and various resistance genes [144]. It is increasingly utilized for detecting carbapenemase-producing Gram-negative bacteria [145,146,147,148]. In this method, carbapenem is incubated with proteins extracted from bacteria for 1–4 h, and the resulting spectra, containing the ionized carbapenem’s charge-to-mass ratio and its breakdown products, are examined to determine the type of carbapenemase and Gram-negative bacteria variation that are involved [82,149,150]. MALDI-TOF can also detect modifications in lipid A. A specific peak of lipid A at m/z value 1796.2 is observed in the spectra [143,151]. Recently, Dortet et al. developed the MALDIxin test, a MALDI-TOF MS-based method capable of detecting lipid A modifications (e.g., pEtN addition) that confer a polymyxin-resistant phenotype to *E. coli* in under 15 min. It also differentiates between chromosome- or plasmid-mediated resistance [152].

### 7.4. Rapid Polymyxin Nordmann Poirel (RPNP) Test for Enterobacteriaceae

Nordmann et al. developed the fast polymyxin NP test which assesses colistin resistance in *Enterobacteriaceae* isolates [137,138]. This test offers affordability, simplicity, and a short turnaround time (<2 h). It relies on a straightforward pH-based mechanism, where colistin resistance is indicated by a color change [143]. Implementation involves two solutions: a rapid polymyxin NP solution and a polymyxin stock solution, both easily prepared with common reagents and storable at −4 °C [153]. Caniaux et al., in their study, suggest combining this test with isolate growth on selective media to ascertain colistin resistance [154]. Extensive validation is needed for non-fermenter colistin-resistant Gram-negative bacteria. A positive result, indicating polymyxin resistance, manifests as a color change to yellow due to bacterial growth in the presence of polymyxin, signifying carbohydrate/glucose metabolism. Conversely, a negative result occurs when the color remains orange, denoting the absence of bacterial growth [5].

### 7.5. Inhibition of MCR-1 Activity

Some of the studies reported that the active catalytic site of the MCR-1 protein contains zinc-binding residues, and zinc ions play a crucial role in determining the phosphoethanolamine transferase activity of MCR-1. This activity governs bacterial resistance to colistin [155]. Deprivation of zinc ions in *E. coli* leads to a reduction in the MIC value of colistin, underscoring the pivotal role of zinc [156]. Building on this principle, several phenotypic detection methods have been developed that utilize either EDTA (ethylenediaminetetra-acetic acid) or DA (dipicolinic acid) to chelate zinc ions, preventing MCR-1’s enzymatic action. Reduced MCR-1 activity renders bacteria susceptible to colistin. Examples of such assays include the MRPNP (Modified Rapid Polymyxin Nordmann Poirel), CMR (Colistin MIC Reduction), CDT (Combined Disc Test), colistin MAC test, and alterations in Zeta potential, among others [157,158]. These chelator-based assays have shown greater efficacy comparing *E. coli* to other Gram-negative bacteria. Therefore, their efficacy warrants validation across different species, particularly *K. pneumoniae* [143].

### 7.6. Polymyxin Drop Test

Initiated for testing defense compounds against isolates of *Brucella*, this test has been refined for routine laboratory use in evaluating resistance to polymyxins. The process entails adding inoculated isolates at 0.5 McFarland to a Mueller–Hinton agar plate, coupled with a single drop of polymyxin solution (16 mg/L) [159]. By diluting the antibiotic powder or employing eluting discs containing polymyxins, the polymyxin solution performs optimally and maintains a concentration of 16 mg/L. After that, plates are incubated for 16 to 18 h at 35 °C following a 15 min rest period at room temperature. After incubation, isolates are characterized by the absence or presence of a zone [5]. In a different investigation, the effectiveness of the Drop Test in identifying *Enterobacterales* and non-fermenting Gram-negative bacilli resistant to carbapenems with polymyxin B resistance was assessed. The Drop Test offers a rapid and straightforward method to detect polymyxin resistance, expediting therapeutic interventions [160].

## 8. Molecular Methods of Detection

The molecular methods are used in complement with the above-described phenotypic methods and help in confirming the resistance status. They are costly and require skills to perform but can yield accurate results in a very short amount of time. The most commonly used molecular methods of detection are described here.

### 8.1. Whole Genome Sequencing (WGS) and Polymerase Chain Reaction (PCR)

The method known as whole genome sequencing (WGS) screens the entire genome, enabling the identification of known and unknown resistance mechanisms within a maximum of 2 days, depending on the instrument used [153]. WGS allowed the discovery of *mcr*-1, the first plasmid-mediated gene granting colistin resistance [15].

PCR is another commonly used method for gene detection. Only one kind of *mcr* gene may be identified with conventional PCR in a given reaction. Numerous PCR variants have been developed [161] to increase the detection of *mcr* genes and variants in a single reaction. These include multiplex PCR and real-time PCR, which uses SYBR Green, probes, or Taqman probes [162].

Nijuhis et al. (2016) developed a real-time PCR approach with 100% accuracy to identify the *mcr*-1 gene in DNA directly extracted from clinical stool samples, with an LOD (Limit of Detection) of 3–30 cfu/reaction [161]. Similarly, With an LOD of 101–108 copies of DNA, Chabou et al. developed a quantitative real-time PCR test using a Taqman probe to identify the *mcr*-1 gene in feces samples with 100% specificity [163]. A quantitative real-time PCR test based on SYBR Green was created by Bontron et al. to detect the *mcr*-1 gene with 100% specificity and a limit of detection of 102 cultivated bacteria [164]. Subsequently, Dona et al. developed a SYBR Green-based assay to detect the *mcr*-1 gene from fecal and cultured samples after suspending the samples in Luria Bertani (LB) enrichment broth and plating on agar plates containing 4 mg/L of colistin [165]. Chalmers et al. (2018) highlighted the impact of incubation time on the detection of small numbers of resistant bacteria, by developing a single real-time PCR technique to detect *mcr*-1 and *mcr*-2 genes in *E. coli* isolated from cecal and fecal samples [166].

In order to identify the *mcr*-1, *mcr*-2, and *mcr*-3 genes in *Enterobacteriaceae* isolated from diverse sources, Li et al. (2017) created a new SYBR Green-based test [167]. Tolosi et al. (2020) expanded the application of real-time PCR to identify the genes *mcr*-4 and *mcr*-5 in addition to *mcr*-1, *mcr*-2, and *mcr*-3 in environmental samples and bacterial isolates [168]. Block multiplex PCR was developed by Rebelo et al. (2018) as a rapid and easy way to identify *mcr* genes and variations (*mcr*-1, *mcr*-2, *mcr*-3, *mcr*-4, and *mcr*-5) [169]. Lescat et al. (2018) created a quick-turnaround multiplex PCR to find the *mcr*-1 through *mcr*-5 genes in a single reaction mix [170].

### 8.2. Loop-Mediated Isothermal Amplification (LAMP)

LAMP is a technique used to amplify nucleic acids under a static temperature condition, unlike PCR which requires temperature cycling. It employs Bst DNA polymerase to synthesize DNA via strand displacement. In under an hour, 10^9^ copies of the target gene were produced, making it rapid and specific [171]. Zou et al. utilized LAMP to detect the *mcr*-1 gene in *Enterobacteriaceae* isolates, assessing results through chromogenic visualization and real-time turbidity monitoring. They found LAMP to be 10 times more sensitive than conventional PCR [172]. Imirzalioglu et al. evaluated the specificity and sensitivity of the commercially available eazyplex SuperBug *mcr*-1 kit by AmplexBiosystems GmbH, Giessen, Germany, finding it to be 100% specific and sensitive, albeit unable to detect the *mcr*-2 gene [173]. Given the existence of multiple *mcr* gene alleles, a single LAMP assay may not provide comprehensive information. Thus, Zhong et al. developed a novel multiplex LAMP assay in 2019, utilizing restriction endonucleases to detect *mcr*-1 to *mcr*-5 genetic variants with 100% specificity and sensitivity [174]. Using primers modified with restriction endonucleases, they constructed a double-LAMP system for *mcr*-2 and 5 and a triple-LAMP system for *mcr*-1, 3, and 4. This allowed them to identify individual *mcr* genes based on the length of fragments and band numbers of digested LAMP-amplified products. Despite many drawbacks, including decreased sensitivity and specificity in cases where the sample contains multiple *mcr* genes, the likelihood of a single strain containing numerous *mcr* genes is low, making this approach applicable in practical scenarios. Moreover, the method’s rapidity, with a total operating time of less than 60 min, and user-friendly nature with no specialized instrument requirements, underscore its potential as a near-patient screening test [5].

### 8.3. Microarray

Microarray technology enables the rapid detection of multiple genes within a short timeframe. In 2017, Bernasconi et al. explored the CT103XL microarray system, a commercially available platform, for detecting various antibiotic resistance genes in bacterial cultures [175]. They examined 106 strains of *Enterobacteriaceae* from environmental, animal, and human sources demonstrating the system’s capability to detect *mcr*-1 (including its variants, *mcr*-1.2 to *mcr*-1.7) and *mcr*-2 genes, as well as β-lactamase genes (class A, B, C, D *bla* genes), with 100% accuracy. The CT103XL microarray system operates on a multiplex ligation detection reaction principle. Probes, comprising sequence-specific arms for target genes, are a binding site for universal primers, and before hybridizing to the microarray, ligation and amplification are performed on a ZIP code. The primer has biotin labeling integrated into it that facilitates probe visualization, which is automatically interpreted by software. Detection using microarray typically takes approximately 6.5 h, with costs ranging from EUR 50 to 85. However, CT103XL cannot identify further *mcr* gene allelic variations, such as *mcr*-3 and *mcr*-4. Nonetheless, the microarray platform offers flexibility for updates to incorporate the detection of emerging antimicrobial resistance genes. Despite its advantages, microarray-based detection has limitations, including its high cost and the expertise required to perform the assay. However, ongoing advancements may mitigate these challenges, enhancing the utility of microarray technology in antibiotic resistance gene detection.

### 8.4. Ribotyping (16S-23S ITS)

A useful method for examining the epidemiology of different harmful bacteria is ribotyping. In *K. pneumoniae*, eight highly conserved operons encode 23S and 16S rRNA on the chromosome. Restriction enzymes (REs) may accurately cut these operons to produce restriction pattern bands that aid in differentiation as well as analysis and interpretation. Ribotyping has emerged as a powerful typing method due to its ability to overcome limitations associated with other traditional typing methods, like inadequate typeability and reproducibility [176]. To address these limitations, molecular techniques employing ribotyping have been developed. For instance, the Digoxigenin-labeled rDNA probe and EcoRI RE have been used to create a ribotyping database of *K. pneumoniae*, removing the requirement for radioactive probes and the hazards they pose [177]. These advancements enhance the accuracy and efficiency of ribotyping, making it an indispensable tool for epidemiological studies and bacterial typing in clinical and research settings.

### 8.5. Multilocus Sequence Typing (MLST)

It is a commonly used DNA sequence-based method for the molecular characterization and genetic relatedness evaluation of different bacterial genera [178,179]. It offers transferable data accessible through global databases, facilitating evolutionary analyses by multiple users. Recently, six sequence categories were revealed when the clinical isolates of enteral and extraintestinal *K. pneumoniae* in China were homologously analyzed using MLST [180], all of which were classified as ST5235. In a study conducted in Taiwan, using Pulsed-Field Gel Electrophoresis, or PFGE, the molecular epidemiology of 43 *K. pneumoniae* isolates was examined, uncovering the transmission of various clones [181]. A number of investigations demonstrated an MDR profile with respect to a wide range of antibiotics, including ciprofloxacin, gentamicin, cephalosporins, and florfenicol. Two unique STs—ST101 and ST258—were also identified with MLST analysis among the 14 *K. pneumoniae* isolates [182].

### 8.6. Infrared Biotyping (IRBT)

Infrared biotyping (IRBT) has emerged as a promising method for typing bacterial strains, particularly in the context of hospital hygiene management [183]. The purpose of the study was to set cutoff value limits and standardize culture procedures for *K. pneumoniae* isolate typing utilizing the IR Biotyper (IRBT) methodology. The method was further contrasted with widely utilized typing techniques including WGS, PFGE, and MLST [184]. Results obtained via IRBT typing closely matched those from PFGE and WGS analyses. Notably, IRBT provides notable benefits in terms of cost-effectiveness and time efficiency. Consequently, IRBT emerges as a highly effective method for typing bacterial strains, facilitating timely investigations into hospital outbreaks with reliability and effectiveness [177].

## 9. Judicial Use of Colistin

**Figure 3:**
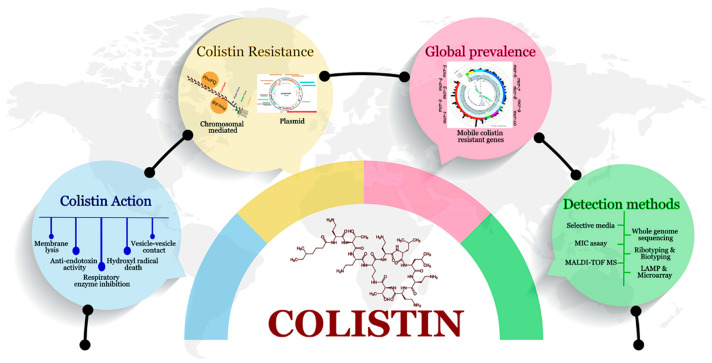
Summary of colistin mechanism of action, resistance, global prevalence, and detection method for better surveillance in Gram-negative bacteria.

The surge in antimicrobial resistance (AMR) among Gram-negative bacteria (GNB) is a pressing concern in the scientific community due to their propensity to cause severe infections and rapid development of resistance to antibiotics. The misuse and overuse of antibiotics in healthcare and agriculture have accelerated the emergence and dissemination of antibiotic-resistant strains. This global issue, acknowledged by the World Health Organization (WHO), is exacerbated by the complex structures of GNB and their capacity to acquire and transfer resistance genes through horizontal gene transfer. While AMR is an inevitable natural phenomenon, its rate has escalated due to the overuse and misuse of antibiotics in the fields of human and veterinary medicine. Antibiotic overuse causes bacteria to experience selection pressure, which accelerates the formation and spread of antibiotic-resistant strains. Initially, the challenge of AMR was predominantly confined to clinical and community settings, with transmission occurring primarily from infected patients. However, recent discoveries highlight how important the environment is to the spread and establishment of resistant strains [185,186,187]. AMR is also widely being observed in agricultural settings, which is again very worrisome [188,189].The environmental hotspots that contain a large number of ARB as well as promote the dissemination of resistant strains are considered to be very critical points for the control of resistance [190]. These hotspots include sites where the untreated effluent from different sources is released directly into the water bodies, e.g., sewage treatment plants, pharmaceutical industries, hospitals, etc. [191]. The other sites that might be contaminated with antibiotic residues can also serve as hotspots and when the bacteria are exposed to a pool of different antibiotics at these sites, they become multidrug-resistant (MDR), and subsequently, many ecological and evolutionary factors play a role in the establishment and further proliferation of these bacteria. The infections caused by such MDR strains, after they are transferred from the environment to humans or animals via different routes, become very difficult to treat. Apart from this, the food colonized by bacteria, air-borne aerosols, etc., can also act as a vector that leads to the transfer of ARB from the environment to human beings and animals [192,193,194]. Due to the lack of availability of drugs in the pipeline against the rapidly emerging deadly antibiotic-resistant GNB, at the present time, different combinations of approaches are being tried. Colistin, a type of polymyxin antibiotic, was previously restricted for treating Gram-negative bacteria (GNB) because of its harmful effects, which include neurotoxicity and nephrotoxicity. However, it is currently being gradually reintroduced to treat infections brought on by bacteria that are resistant to drugs (MDR). GNB therapy with colistin is considered a last resort within prescribed limits. However, the fact that GNB is utilizing a plethora of mechanisms with the recently discovered plasmid-mediated mode to develop and transfer resistance against polymyxin (colistin) is very alarming, which could ultimately lead to pan-resistance. Therefore, the need of the hour is the adoption of a holistic and pragmatic approach with sound management strategies including the proper discharge of wastewater, and proper treatment strategies to deal with MDR bacterial infections to avoid therapeutic impasses. The concept of ‘One health approach’ is being emphasized by several researchers, wherein it has been said that there are connections in each other’s health between the environment, animals, and human health, and if there is any misuse of antibiotics in any of these settings, it will have adverse effects [195,196,197]. To ensure antibiotic effectiveness, an integrated approach should be developed with considerations for human and animal well-being [187,198]. Colistin treatment must be administered only after proper antimicrobial susceptibility testing and under strict supervision. Addressing antimicrobial resistance (AMR) demands a multidisciplinary approach, encompassing strategies like developing novel drugs, repurposing existing medications, exploring drug combinations, and promoting the judicious use of current drugs. Among them, the combination of colistin and other antibiotics was found to be effective against colistin-resistant GNB, commonly called combination therapy. Studies have reported that colistin combined with various antibiotics, including rifampin, azithromycin, fusidic acid, linezolid, ancomycin, aztreonam, ceftazidime, imipenem, and tigecycline, have effective synergistic activity against colistin-resistant GNB [199,200,201]. Sukrit Srisakul et al. reported that colistin in combination with sulbactam showed effective synergistic activity with a synergy rate of 86.7% against colistin-resistant *A. baumannii* as compared to colistin alone [202]. Beyond in vitro, colistin with clarithromycin combination therapy has been shown to be effective in clinically relevant doses against colistin-resistant *K. pneumoniae*-harboring *mcr*-1 in murine thigh infection [203]. So, in the future, an effective combination of colistin with various antibiotics may be useful as an alternative therapy to combat colistin-resistant GNB. By employing these measures collectively, we can mitigate the threat of AMR and combat this global health crisis effectively. Figure 3 depicts the comprehensive understanding of the colistin antimicrobial drug in GNB for better surveillance and strategy in the future.

## 10. Conclusions

In conclusion, the escalating antimicrobial resistance (AMR) seen in Gram-negative bacteria (GNB) poses a significant global health risk, largely attributed to the improper and excessive use of antibiotics in both medical and agricultural settings. The emergence of antibiotic-resistant strains, coupled with their spread through environmental hotspots, emphasizes the urgent need for comprehensive management strategies. Colistin, previously restricted due to adverse effects, is now being reintroduced as a last-resort treatment for multidrug-resistant (MDR) GNB infections. However, the concerning increase in colistin resistance, facilitated by various mechanisms including plasmid-mediated transfer, underscores the importance of rigorous antimicrobial stewardship and effective wastewater management.

Innovative approaches are desperately needed to overcome the resistance to colistin. It is important to conduct research on alternative therapies, such as novel antibiotics and medicinal combinations. Slowing the development and spread of resistance also requires supporting antimicrobial stewardship initiatives to guarantee the prudent use of colistin and other antibiotics. Furthermore, it is critical to invest in surveillance systems that can monitor resistance trends and identify emerging threats before they become serious. Using a One Health strategy can offer a complete framework for managing colistin resistance as it incorporates activities from the human health, animal health, and environmental sectors. Collectively executing these strategies will allow us to effectively address AMR and protect public health for future generations.

## Data Availability

Not applicable.
